# How I personalize fluid therapy in septic shock?

**DOI:** 10.1186/s13054-023-04363-3

**Published:** 2023-03-24

**Authors:** Xavier Monnet, Christopher Lai, Jean-Louis Teboul

**Affiliations:** AP-HP, Service de Médecine Intensive-Réanimation, Hôpital de Bicêtre, DMU 4 CORREVE, Inserm UMR S_999, FHU SEPSIS, CARMAS, Université Paris-Saclay, 78 Rue du Général Leclerc, 94270 Le Kremlin-Bicêtre, France

**Keywords:** Passive leg raising, Tidal volume, Fluid challenge, Volume expansion, Cardiac output, Fluid balance

## Abstract

During septic shock, fluid therapy is aimed at increasing cardiac output and improving tissue oxygenation, but it poses two problems: it has inconsistent and transient efficacy, and it has many well-documented deleterious effects. We suggest that there is a place for its personalization according to the patient characteristics and the clinical situation, at all stages of circulatory failure. Regarding the choice of fluid for volume expansion, isotonic saline induces hyperchloremic acidosis, but only for very large volumes administered. We suggest that balanced solutions should be reserved for patients who have already received large volumes and in whom the chloremia is rising. The initial volume expansion, intended to compensate for the constant hypovolaemia in the initial phase of septic shock, cannot be adapted to the patient’s weight only, as suggested by the Surviving Sepsis Campaign, but should also consider potential absolute hypovolemia induced by fluid losses. After the initial fluid infusion, preload responsiveness may rapidly disappear, and it should be assessed. The choice between tests used for this purpose depends on the presence or absence of mechanical ventilation, the monitoring in place and the risk of fluid accumulation. In non-intubated patients, the passive leg raising test and the mini-fluid challenge are suitable. In patients without cardiac output monitoring, tests like the tidal volume challenge, the passive leg raising test and the mini-fluid challenge can be used as they can be performed by measuring changes in pulse pressure variation, assessed through an arterial line. The mini-fluid challenge should not be repeated in patients who already received large volumes of fluids. The variables to assess fluid accumulation depend on the clinical condition. In acute respiratory distress syndrome, pulmonary arterial occlusion pressure, extravascular lung water and pulmonary vascular permeability index assess the risk of worsening alveolar oedema better than arterial oxygenation. In case of abdominal problems, the intra-abdominal pressure should be taken into account. Finally, fluid depletion in the de-escalation phase is considered in patients with significant fluid accumulation. Fluid removal can be guided by preload responsiveness testing, since haemodynamic deterioration is likely to occur in patients with a preload dependent state.

## Background

The first therapeutic measure in septic shock, volume expansion, is seemingly simple: a few hundred millilitres of fluid injected intravenously over a few minutes. However, nothing is simple in the strategy of using fluids in septic patients. Several different types of fluids are available today [1]. The effects of a fluid bolus are inconsistent and transient [2]. Fluids are risky treatments, particularly during septic shock [3]. Then, many questions raise in practice. Which fluid to choose? Should it be infused? How to evaluate its risk and refrain from infusing it? How to predict its effects? How to evaluate them?

To these questions, one can provide a standard and unequivocal answer. This is what the current guidelines do [4], without distinguishing according to the clinical context and the patient characteristics. The aim is likely to make them general enough to be applied widely, which is undoubtedly laudable. Another reason is that these guidelines are based on existing randomized controlled trials, which have often paid little attention to the particularities of patients they included. So, while these recommendations are of great value, there is probably room for a more personalized fluid management [5, 6]. Contrary to a “universal” strategy, the idea that “one size does not fit all” has been defended for many years about several strategies in critical care medicine [7, 8]. The need for personalization of treatment has emerged as a virtuous goal to pursue [5], especially in septic shock [6, 9]. This is the case for fluids as for any other treatment.

In this review, we will describe what a personalized fluid strategy in septic shock can be, based not only on the existing publications, but also on the pathophysiological concepts underlying the effects of fluids. From the choice of fluid type to the decision to remove the fluid administered in excess, we will try to show that, at each stage, the choices can reasonably be individualized.

### How to customize the choice of fluid type?

#### No colloids, but balanced crystalloids…

In septic shock, crystalloids are indicated in first intention by the Surviving Sepsis Campaign (SSC) [4]. However, even though few evidence supports this choice, iso-oncotic albumin may be chosen for instance in patients with low albuminemia and perhaps with fluid accumulation, to increase the plasma oncotic pressure.

The current debate is mainly between the use of balanced or unbalanced crystalloids. Using saline as the main fluid source can induce hyperchloremic acidosis, which may decrease kidney cortical perfusion and could induce renal failure. Several studies have suggested that compared to chloride-rich solutions, balanced solutions reduce the incidence of renal failure [10, 11] and possibly mortality [11–13].

Accordingly, the 2021 SSC guidelines suggested to prefer balanced crystalloids in all septic shock patients [4]. This weak recommendation is mainly based on the SMART study, which had its own limitations (a single centre, no individual randomization and questionable identification of sepsis) [13].

#### Balanced crystalloids, but for all patients?

**Fig. 1:**
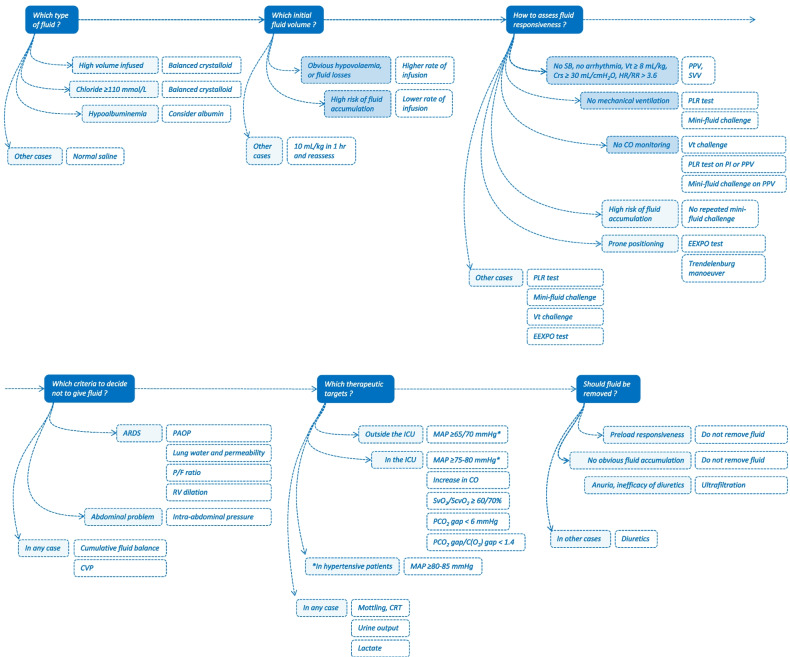
Criteria to customize the fluid strategy at different steps of septic shock. *ARDS* acute respiratory distress syndrome, *CO* cardiac output, *C(O*_*2*_*)* oxygen content, *CRT* capillary refill time, *CVP* central venous pressure, *EEXPO* end-expiratory occlusion, *HR* heart rate, *ICU* intensive care unit, *MAP* mean arterial pressure, *P/F ratio* ratio of the arterial oxygen partial pressure to the inspired oxygen fraction, *PAOP* pulmonary artery occlusion pressure, *PCO*_*2*_ carbon dioxide partial pressure, *PLR* passive leg raising, *PPV* pulse pressure variation, *PI* perfusion index, RR: respiratory rate, *RV* right ventricular, *SvO*_*2*_ mixed, venous oxygen saturation, *ScvO*_*2*_ central venous oxygen saturation, *SVV* stroke volume variation, *Vt* tidal volume

Should we favour these balanced solutions as first intention in all septic shock patients? Like other authors [6], we think that this choice should rather be personalized. Normal saline may induce hyperchloremia [14], but only if large volumes are administered. In a 70-kg patient, 12 litres must be infused for the blood bicarbonate level to drop by 10 mmol/L [15]. Balanced crystalloids should be logically reserved for patients requiring large fluid volumes and in whom chloremia rises significantly [16] (Fig. 1). Moreover, unbalanced crystalloids must be preferred in case of traumatic brain injury [17] and hypochloremia. In any case, chloremia should be measured to tailor the type of fluids [16].

Indirectly, this opinion is reinforced by the results of the PLUS study [18]. Previous studies had demonstrated a beneficial [11] or neutral [19, 20] effect of balanced crystalloids compared to 0.9% saline. The PLUS study compared PlasmaLyte *vs.* normal saline in critically ill patients (not only septic) [18]. The study was negative: mortality and risk of acute renal failure were not lower with the balanced crystalloid, including in septic patients. But is this surprising? The study included an indistinct population of intensive care unit (ICU) patients, who received <2 litres of fluid per day during the first 2 days. Then, chloremia was ultimately very little different between groups [18], so that the balanced solution could not exert any benefit in the treated group. The study may have been positive if the use of balanced solution had been conditioned to the infused volume and chloremia.

### We must personalize the fluid balance of patients in septic shock!

#### Fluid is harmful... but studies that tested fluid restriction showed no effect on mortality

On the one hand, fluid accumulation during the ICU is harmful [2]. Numerous studies have demonstrated that the cumulative fluid balance independently influences mortality in ICU patients [21], particularly during septic shock [3] and acute respiratory distress syndrome (ARDS) [22].

On the other hand, the several interventional studies that compared “liberal” vs. “conservative” or “restrictive” fluid strategies [23–30] provided consistent results: such strategies reduce the fluid balance, without deleterious effects, especially on the kidneys, but with no reduction in mortality, even if this was not the primary objective of some studies [25–27]. Some showed that the reduction in fluid balance decreased the duration of mechanical ventilation and the ICU stay [28, 29].

#### … but they have two major flaws

**Fig. 2:**
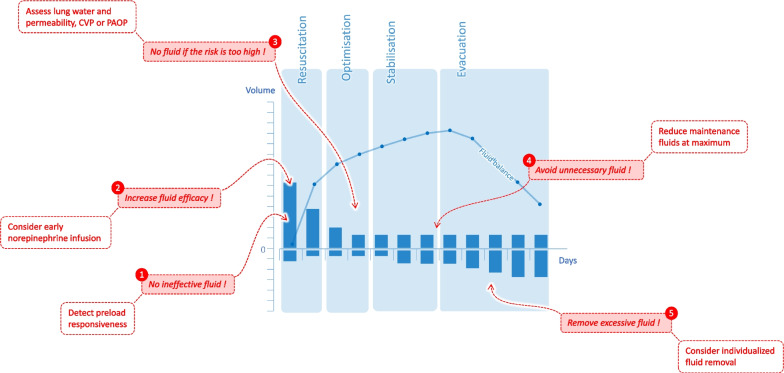
Means for reducing the cumulative fluid balance depending on the stage of resuscitation of septic shock. *CVP* central venous pressure*, PAOP* pulmonary artery occlusion pressure

First, a maximal reduction of the fluid balance can only be achieved if all possible means are used for this purpose (Fig. 2). However, studies have restricted either resuscitation fluids [24, 26, 30, 31], or maintenance and replacement fluids [26, 27, 31], or protocolized fluid removal [27], but never all three at the same time. In the CLASSIC study, fluid restriction was limited to fluid bolus infusion [25]. Then, the total volume administered was little different between groups (at day-5, only 700 mL saved in the restrictive group [25]), reducing the chances of observing a difference in outcome. Conversely, in the RADAR-2 study, a strategy based on limiting maintenance fluids and removing fluid reduced the cumulative fluid balance to a larger extent (3 300 mL at day-5) [27].

The second shortcoming is that these studies did not individualize the strategy according to the patient characteristics. Some have included patients particularly at risk of fluid accumulation, such as ARDS patients [28]; others have been conducted in an indistinct population [27], including patients in whom the expected benefit of a conservative strategy was necessarily less. Also, no study has tested an individualized fluid restriction on the physiological state of the patient. For instance, preload responsiveness has not been considered while this is known to reduce the fluid balance [32, 33].

Thus, if the reduction of the cumulative fluid balance seems to be a goal to pursue in septic shock patients, the strategy to achieve it should not be standardized. We suggest a method which considers all the resuscitation phases (Fig. 2) and which is personalized (Fig. 1).

### How to customize the initial fluid volume?

The latest SSC guidelines state that septic patients with hypotension or an elevated blood lactate should receive ≥30 mL/kg of crystalloid within 3 hours of presentation [4]. This volume, which is only based on an observational study [34], aims to compensate for relative and absolute hypovolaemia during initial septic shock. The patient’s weight is the only parameter of individualization. Yet, the degree of initial volume deficit obviously differs among patients. First, relative hypovolaemia is linked to venous vasodilation, depending on shock severity. Second, in some patients, fluid losses may account for absolute hypovolaemia, which must also be compensated. For example, the fluid deficit must be larger during peritonitis than during pneumonia. The recommendation of an arbitrary volume of 30 mL/kg inevitably leads to under-resuscitation in some patients and fluid overload in others [35]. In addition, tolerance to fluid administration also depends on the cardiac function. The initial fluid therapy should then be individualized. For instance, deciding to reduce the initial volume infused only based on the patient condition may increase mortality [34].

In the very initial phase, as hypovolaemia is constant, testing preload responsiveness is not warranted and may delay resuscitation. Afterwards, volume expansion should be individualized according to the degree of preload responsiveness, to avoid unnecessary fluid infusion (Fig. 1) [36]. This is recommended by the SSC which, on this point, suggests individualization of treatment [4]. Assessing preload responsiveness should not be delayed as it disappears quickly after the onset of septic shock and it [37, 38]. In the ANDROMEDA-SHOCK study, after having received 27 mL/kg of fluids, 70% of patients were still preload responsive at inclusion, and among them, <20% remained so four hours later [38]. Guiding initial fluid therapy by evaluating preload responsiveness during septic shock can reduce the fluid balance [32].

### How to customize the choice of test or index of preload responsiveness?

Reviews detailing indices and tests to detect preload responsiveness can be found elsewhere [39]. Our goal here is to describe what leads to choosing one over another. This choice depends on three questions (Fig. 1).

#### Is the patient intubated?

The passive leg raising (PLR) test and the mini-fluid challenge do not require mechanical ventilation. This makes them usable, for example, in the emergency department, while patients are not yet intubated [39]. Of course, tests based on cardiopulmonary interactions can only be used in intubated and ventilated patients. Pulse pressure variation (PPV) and stroke volume variation (SVV) (in absolute value) can only be used in patients without spontaneous breathing, with a tidal volume (Vt) ≥8 mL/kg, with a compliance of the respiratory system ≥30 mL/cmH_2_O, without intra-abdominal hypertension, with sinus cardiac rhythm and with a ratio of heart rate to respiratory rate ≥3.6 [40], i.e. in a minority of shock patients [39]. The changes in diameter of inferior and superior vena cava have the same limitations (except arrhythmia and for superior vena cava, the high respiratory rate). Even under optimal conditions of use, it has a lesser diagnostic value, especially for the inferior vena cava distensibility [41].

The end-expiratory occlusion (EEXPO) test (end-expiratory pause of 15 seconds, which increases cardiac output (CO) in the event of preload responsiveness [42]), can be used in ventilated patients even in case of arrhythmia, whatever the Vt and the positive end-expiratory pressure level. However, the patient must tolerate this rather long breathing interruption [43].

In ventilated patients with a Vt of 6 mL/kg, the Vt challenge (temporary increase in Vt to 8 mL/kg, which increases PPV in case of preload responsiveness [44]) is valid with spontaneous breathing, which makes it widely usable. Its validity is better and better demonstrated [39]. Recruitment manoeuvres [45] are only indicated in ARDS patients and not routinely [46], which greatly limits their use. Finally, in prone positioning, the Trendelenburg manoeuvre may be used to transfer blood to cardiac cavities and detect preload responsiveness [47]. It has received little validation at the moment. In prone position, the Vt challenge is also valid [48] (Fig. 1).

#### Which haemodynamic monitoring is in place?

The great advantage of the Vt challenge [44], the PLR test [49, 50] and perhaps the mini-fluid challenge [51], is that they can be carried out with the simple PPV, i.e. only requiring a single arterial line. For the PLR test, many other variables can replace CO, such as end-tidal carbon dioxide in ventilated patients [52], or the perfusion index of plethysmography [53, 54].

The EEXPO test requires a precise measurement of CO, because the changes in stroke volume induced are small [42]. If echocardiography is used, end-expiratory and end-inspiratory pauses should be combined [55] to amplify the changes in velocity time integral and to make them more compatible with the precision of echocardiography [56].

#### What is the risk of fluid accumulation?

The mini-fluid challenge requires the injection of 100-150 mL of fluid, which cannot be withdrawn if ineffective [57]. It is then reasonable not to repeat it if the risk of fluid accumulation is high. From this point of view, it is probably better suited to the operating room than the ICU (Fig. 1).

### How to personalize the decision to add a vasopressor to fluid?

#### Vasopressors may have synergistic effects with fluids

Norepinephrine is usually started once fluid boluses no longer exert any benefit, to increase arterial pressure [4]. However, its effects on systemic venous return should not be overlooked, as they may contribute to limiting the fluid balance during septic shock (Fig. 2). By binding to the venous alpha-adrenergic receptors, norepinephrine induces vasoconstriction which increases the part of stressed blood volume [58]. It increases mean systemic pressure, exerting a fluidlike effect [59]. In addition, volume expansion performed under vasopressor may exert a larger effect on cardiac preload, because it occurs in a constricted venous territory [60].

#### Vasopressors may be administered early in some patients

These venous effects of norepinephrine are synergistic with the administration of fluid, which may reduce the quantity of fluid administered for resuscitation (Fig. 2). Observational studies in septic patients suggested that an early start of norepinephrine could reduce the fluid balance [61, 62], and one of them matching patients with a propensity score suggested a better survival when norepinephrine was administered very early [63]. However, opposite observational results have been reported [64].

The CLOVERS study randomized patients between a restrictive strategy, prioritizing lower intravenous fluid volumes and vasopressors, and a liberal strategy, prioritizing higher fluid volumes before starting vasopressors. Although less fluid was administered within the first 24 hours, mortality at day-90 did not differ between groups [31]. However, the interventional arm combined early norepinephrine administration with a restrictive fluid strategy, suffering from the flaws of the studies mentioned above, so the prognostic impact of early norepinephrine administration alone cannot be inferred from these results.

Moreover, in this study, patients in whom norepinephrine was administered earlier were not selected [31]. This early administration of norepinephrine should likely be considered in the most hypotensive septic patients, to prompt blood pressure restoration. Also, norepinephrine is likely most powerful in patients with marked vasodilation, as indicated by a low diastolic pressure (e.g. <40 mmHg) (Fig. 1). The diastolic shock index (heart rate divided by diastolic arterial pressure), which predicts death better than each variable alone, may also be considered for this purpose [65].

In addition, the effectiveness of a fluid bolus on CO is ephemeral. In the perioperative context, the effects on CO of a 250-mL crystalloid bolus were shown to disappear after 10 minutes [66]. This duration could be even shorter in septic patients, due to vasodilation and capillary leakage. Even if it has not been investigated, the effects of a fluid bolus on microcirculation are also likely transient. Therefore, in septic patients, restoring haemodynamics with norepinephrine probably allows a more persistent effect, although this has not been proved yet.

### How to customize the criteria for stopping fluid infusion?

Good fluid management should include safety criteria, indicating that the risk of fluid infusion outweighs the expected benefit [67]. These criteria are particularly important in the stabilization phase, after having corrected the initial hypovolaemia [67] (Fig. 2). Two clinical situations may justify monitoring of particular indices.

#### In patients with ARDS

In these patients, to alert to the risk of additional fluid infusion, one often uses the ratio of arterial partial pressure of oxygen (PaO_2_) to fractional inspiration of oxygen (FiO_2_). However, it can also be lowered by pulmonary consolidations, shunt related to microthrombi, etc., which are not influenced by additional fluid infusion.

“Sophisticated” haemodynamic monitoring techniques estimate the pulmonary risk of fluid resuscitation more directly. The pulmonary artery catheter estimates the pulmonary capillary pressure, which propels fluid into the interstitium. The estimation of this pressure by echocardiography is poorly reliable in critically ill patients [68].

Extravascular lung water and pulmonary vascular permeability measured by transpulmonary thermodilution directly reflect the risk of fluid leakage towards the interstitium and alveoli [69]. The maximum value of lung water influences the prognosis of ARDS patients, independently of other severity criteria [70].

Lung ultrasound evidences interstitial lung oedema by B-lines [71]. While reliable for diagnosing interstitial oedema, its value for quantifying the total volume of pulmonary oedema can only go through a tedious count of the B-lines number [72]. Moreover, it neglects alveolar oedema, so that it poorly reflects total lung water [73].

#### In patients with intra-abdominal hypertension

Especially in patients with abdominal problems, the intra-abdominal pressure (IAP) should be considered to estimate the risk of fluid accumulation which, *via* visceral and parietal oedema, increases IAP [74]. Intra-abdominal hypertension impairs the perfusion of abdominal organs, mainly the kidneys, and independently influences the prognosis of ICU patients [75]. This variable must be considered before deciding to administer a fluid bolus during the stabilization phase [76].

#### Don't forget CVP!

Regardless of the patient, even if CVP does not predict preload responsiveness, its measurement provides a great deal of information that is often overlooked [77]. CVP is the backward pressure of organ blood flow [78], and increased CVP levels are associated with organ dysfunction [79]. Limiting the increase in CVP may be a reasonable goal, and it might be justified to target higher mean arterial pressure targets in patients with elevated CVP.

### How to personalize therapeutic targets?

Fluid boluses are aimed at increasing mean systemic pressure, cardiac preload, consequently stroke volume and CO, and ultimately at improving tissue oxygenation. However, the therapeutic targets must be adapted to the available haemodynamic monitoring and to the context.

#### Outside the ICU

When fluid therapy is conducted in the wards, in the pre-hospital or in the emergency department, the clinical examination must look for clinical signs of improvement in tissue perfusion: disappearance of skin mottling, shortening of capillary refill time, which disappears quite faster and might change even after a single fluid bolus [80, 81]. Improved organ function, especially increased diuresis, is rarely available because of the very short observation time (Fig. 1). Although these signs should always be looked for, they poorly reflect CO changes [82].

The primary effect of a bolus of fluid is to increase CO, and arterial pressure increases only incidentally [83]. Of note, the target of mean arterial pressure may be adapted to the existence of previous hypertension [84]. However, pulse pressure is the one that should be observed to monitor CO changes, because it is the pressure value that best reflects stroke volume. Changes in mean arterial pressure are attenuated, as the sympathetic system tends to keep it constant as CO varies. It is surprising to see that restoring mean arterial pressure can be considered as the objective of fluid therapy [85, 86].

However, even changes in pulse pressure imperfectly reflect CO [83, 87, 88], likely even more when assessed through a brachial cuff. Similarly, the decrease in heart rate is unreliable to detect the fluid-induced increase in CO [88]. This is what justifies measuring CO directly, when possible, to better assess the fluid effects.

#### In the ICU

More variables are available to estimate fluid efficacy. CO is easily measured, either by transpulmonary thermodilution, pulmonary arterial catheter, recommended in the most severe patients [89], or by echocardiography. If no direct measurement of CO is available but an arterial line is in place, a decrease in PPV reflects the decrease in preload responsiveness and demonstrates the response to fluid [90]. In intubated and perfectly stably ventilated patients, the increase in end-tidal carbon dioxide parallels the increase in CO [52, 91]. The amplitude of the plethysmography signal, quantified by the perfusion index, could also reflect the fluid-induced increase in CO, as has been suggested by a few studies [53, 54, 92].

Also, the effects on tissue oxygenation must be assessed [67], as an increased CO does not always result in an improved oxygen consumption, either because oxygen consumption and delivery are independent [93] or because the microcirculation is deranged [94]. In the ICU, several variables are available for this purpose. The reduction of lactate, which is associated with a favourable prognosis [95], is a valid therapeutic objective. Through the central venous catheter which is very often inserted, venous oxygen saturation and carbon dioxide-derived variables [96] show improved tissue oxygenation. These variables are more reliably measured from mixed venous blood if a pulmonary artery catheter is in place. In cases where venous oxygen saturation is normal, the ratio of the veno-arterial difference in carbon dioxide partial pressure to the arteriovenous difference in oxygen content, an estimate of the respiratory quotient, has been suggested as a marker of anaerobic metabolism with faster response than lactate and less false positives [96]. Its normal value has been reported to be <1.4 [97], though different thresholds have been reported [98].

### How to customize fluid depletion?

One of the ways to reduce the total fluid balance is to undertake active fluid depletion, at the “evacuation” phase of the resuscitation–optimization–stabilization–evacuation (ROSE) concept (Fig. 2). Indeed, the reduction of resuscitation fluids alone is insufficient, because maintenance and replacement fluids represent a large proportion of the fluids infused in the ICU [99]. The modalities for this de-escalation are variable [100], and they should be personalized by considering usefulness and safety.

#### In which patients should fluid removal be undertaken?

Fluid depletion should be the most beneficial in patients with the largest fluid accumulation, as indicated by the variables mentioned above: high left ventricular filling pressure, high CVP, very lung water, signs of pulmonary congestion on CT scan and elevated IAP. The presence of soft tissue oedema does not necessarily have to be required, as they may on the contrary be accompanied by a depleted intravascular sector.

Conversely, if fluid accumulation is low, for example in easily resolving septic shock, the benefit of fluid depletion is less obvious. The risk of undertaking it must therefore be weighed against the possible benefits.

#### How to remove fluid?

Diuretics are used first. However, in case of oliguria or anuria, ultrafiltration during renal replacement therapy is the alternative solution [101]. In case of low albumin, 20% hyperoncotic albumin could induce a synergistic effect on fluid removal. In patients ventilated for acute lung injury, a strategy associating positive end-expiratory pressure adjusted to IAP followed by infusion of hyperoncotic albumin and furosemide resulted in negative cumulative fluid balance and decreased lung water and IAP [102].

#### How to choose the dose of fluid to withdraw?

The risk of too much fluid withdrawal is to alter the haemodynamic state. To ensure the safety of fluid depletion, physiological conditions must be considered, which is rarely done in practice [100]. First, the haemodynamic state must be stable, and the vasopressors must be at low dose or stopped.

Then, decreasing cardiac preload with fluid removal can only alter the haemodynamic condition in case of preload responsiveness. Conversely, if ventricles are preload unresponsive, fluid removal must be well tolerated. One should condition the decision to withdraw fluid to the existence of a preload *un*responsiveness. In a study in critically ill patients in a stabilized phase of shock, fluid removal undertaken by ultrafiltration did not induce intra-dialytic hypotension in patients who did not have preload responsiveness, as well as proved by a negative PLR test before depletion [103] (Fig. 1). Even if at the priming of the extracorporeal circuit, a decrease in CO is much more frequent in the case of preload responsiveness than in the case of preload unresponsiveness [104], other factors may be responsible for intra-dialytic hypotension. However, the fact remains that the existence of a preload responsiveness must urge not to withdraw additional fluid.

## Conclusion

Despite its apparent simplicity, fluid therapy is complex. Fluids are drugs, inconsistently effective and endowed with notable deleterious effects. Like any medicine, they must be administered at the right dose and only to patients who need them. To guide fluid therapy, rather than relying on an identical strategy for all patients, it is possible to personalize decisions. Consider the characteristics of the patient (the severity of the vasodilation, the importance of the fluid accumulation), his/her clinical history (to assess the depth of the initial hypovolaemia), his/her physiological conditions (in particular preload responsiveness), should logically ensure effective and safe fluid delivery and removal. If these individual criteria are not considered, clinical studies investigating the various fluid therapy strategies are and will remain negative.

## Data Availability

Not applicable.
